# Periodic pattern detection in sparse boolean sequences

**DOI:** 10.1186/1748-7188-5-31

**Published:** 2010-09-10

**Authors:** Ivan Junier, Joan Hérisson, François Képès

**Affiliations:** 1 Institut des Systèmes Complexes Paris Île-de-France, 57-59 rue Lhomond, F-75005, Paris, France; 2 Epigenomics Project, Genopole, CNRS UPS3201, UniverSud Paris, University of Evry, Genopole Campus 1 - Genavenir 6, 5 rue Henri Desbruères - F-91030 EVRY cedex, France

## Abstract

**Background:**

The specific position of functionally related genes along the DNA has been shown to reflect the interplay between chromosome structure and genetic regulation. By investigating the statistical properties of the distances separating such genes, several studies have highlighted various periodic trends. In many cases, however, groups built up from co-functional or co-regulated genes are small and contain wrong information (data contamination) so that the statistics is poorly exploitable. In addition, gene positions are not expected to satisfy a perfectly ordered pattern along the DNA. Within this scope, we present an algorithm that aims to highlight periodic patterns in sparse boolean sequences, i.e. sequences of the type 010011011010... where the ratio of the number of 1's (denoting here the transcription start of a gene) to 0's is small.

**Results:**

The algorithm is particularly robust with respect to strong signal distortions such as the addition of 1's at arbitrary positions (contaminated data), the deletion of existing 1's in the sequence (missing data) and the presence of disorder in the position of the 1's (noise). This robustness property stems from an appropriate exploitation of the remarkable alignment properties of periodic points in solenoidal coordinates.

**Conclusions:**

The efficiency of the algorithm is demonstrated in situations where standard Fourier-based spectral methods are poorly adapted. We also show how the proposed framework allows to identify the 1's that participate in the periodic trends, i.e. how the framework allows to allocate a *positional score *to genes, in the same spirit of the sequence score. The software is available for public use at http://www.issb.genopole.fr/MEGA/Softwares/iSSB_SolenoidalApplication.zip.

## Background

There is increasing evidence that the organization of the genome plays a crucial role in the interplay between genetic regulation and chromosome structure. At the smallest scale, several experimental studies have highlighted the importance of the positions of the transcription factor binding sites in the functioning of small transcriptional regulatory networks [1-3]. At a larger - but still local - scale, in bacteria many transcription units are known to be located along the DNA close to the gene that encodes their regulating transcription factors [4-6]. At the global scale of the chromosome, both in *Escherichia coli *and in *Saccharomyces cerevisiae*, it has been previously realized that the genes that are regulated by the same transcription factor have a tendency to be periodically spaced along the DNA [7,8]. Recently, the relative positions of phylogenetically conserved gene pairs were also shown to tend to periodically organize along the DNA in *E. coli *[9]. Such periodic organization has been proposed to be responsible for the spatial co-localization of co-regulated genes [10]; indeed, a periodic ordering along the DNA of distal binding sites that can be cross-linked by a bivalent transcription factor (or a larger complex), just as in the case of the *lac *operon or of the λ bacteriophage repressor, leads to a quick and homogeneous formation of transcription factories [11].

More generally, in any kind of signals, the presence of periodic regularities reveals an underlying notion of order. As such, this can provide hints about the signal genesis and/or a base for a further processing of the information, just as in crystallographic experiments. However, the detection of periodic patterns can be drastically hampered by signal distortions [12,13]. Specific techniques, which depend on the nature of the signal, therefore need to be developed - see *e.g. *[14,15] in the context of gene expression data. In this article, we present a method to detect periodic patterns in *boolean sequences, i.e*., the signal *X*(*l*) is a one-dimensional signal that takes values in {0, 1}, the coordinate *l *is discrete and takes values in ℕ. More particularly, we address the question of *sparse sequences*, that is the ratio of the number of 1's to 0's is much smaller than 1. A prototypic example concerns the organization of genes along DNA. For instance, the human genome contains approximately 3 × 10^4 ^genes that are distributed along a 3 × 10^9 ^base-pair long DNA - in this case, *l *stands for the position of the base-pairs forming the DNA. Hence, the ratio 1/0 is on the order of 10^-5^.

One of the major difficulties of periodic detection, especially in the case of sparse data, lies in the robustness of the method with respect to noise, data contamination and missing data. Noise leads to positions of 1's that are different from the ideal periodic case. This is a ubiquitous source of signal distortion since perfect periodic patterns stem from specific types of phenomena, *e.g*. the ordering of atoms in crystals. Data contamination, often referred to as false positives, refers to the points {*l, X*(*l*) = 1} that come from wrong information. Such contamination is commonplace in bioinformatics, especially when predicting features using datasets that are built from genome-wide experiments [16]. Preventing it mostly leads to missing true findings (missing data), that is *X*(*l*) = 0 for values of *l *such that *X*(*l*) should be equal to 1, which is often referred to as false negatives. As a result, datasets may contain both false positives and false negatives - they always do in datasets coming from high-throughput biological experiments [16].

Within this scope, we present a periodic pattern detection method that is particularly robust with respect to noise, data contamination and missing data. The method has two facets, namely, i) it highlights the presence of periodic patterns and ii) it identifies the points that participate in the periodic trends, which are discussed in the two next sections. As an illustration, using both artificial and real datasets, we then show the limitations of standard Fourier-based spectral methods in situations where the present tool is fully efficient.

### Highlighting periodic regularities in boolean sequences

**Figure 1:**
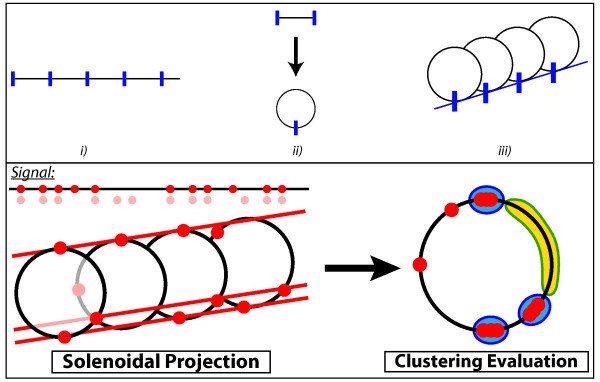
**Principle of the solenoidal coordinate method**. *Upper panel*: The construction of the *P*-periodic solenoids is based on three steps: *i*) the signal support is divided into segments of length *P*, *ii*) the segments are converted into circles, *iii*) the circles are aligned with respect to the locking up points. *Lower panel*: A set of sites (in red, upper left corner) come from a pattern that periodically repeats at some period *P *(blurred red points). Some of the initial periodic sites are missing (false negative, missing data) or have different positions (noise), and random sites have been added (false positive, contaminated data). The position of the sites in a solenoidal coordinate of period *P *(lower left panel) reveals some alignment properties along the solenoid axis. A projection upon the face view of the solenoid converts the alignments into clustered points (rightmost panel). The *SCM *scoring function aims at rewarding both dense regions (indicated in blue) and poor regions (indicated in yellow).

We shall consider a boolean sequence *X*(*l*) of length *L *so that *l *∈ {0, ..., *L *- 1} - *e.g*., in the case of gene positions, *l *stands for a base-pair coordinate and *L *for the length of the genome. We call *N *the number of points {*l*, *X*(*l*)} such that *X*(*l*) = 1 (*e.g*. the number of genes). For the sake of simplicity, in the following, these points will be referred to as *sites*. Our periodic pattern detection method relies on the fact that the sites that are periodically organized according to a period *P *tend to align when the coordinate *l *is wrapped around a *P*-periodic solenoid - the solenoids are built as follows: first, the signal support is divided into segments of length *P*; second, the segments are converted into circles (perimeter = *P*); third, the circles are aligned with respect to the locking up points (Fig. 1). In turn, the site alignments lead to clustering tendencies after a projection onto the face view of the solenoid (Fig. 1). Interestingly, this clustering tendency, or equivalently the tendency for sites to align along the solenoid axis, remains largely unaffected by a small amount of disorder in the positions (noise), by site deletions (false negatives, missing data) or by addition of sites at locations out of phase with respect to the periodicity (false positives, contaminated data). As a result, the presence of a *P*-periodic motif can be efficiently detected by using a scoring function that reflects the good clustering properties of the projected sites along the face view of the *P*-periodic solenoid - hence, the method has been called the solenoidal coordinate method (*SCM*). In particular, such a method is expected to be robust towards strong signal distortions, as we shall see below. The solenoidal picture is useful to have an intuitive (geometric) understanding of the method. From a formal point of view, a site at position *x *leads to a position *x^P ^*on the face view of the *P*-periodic solenoid, which is simply given by the congruence modulo *P, i.e. × *≡ *x^P ^*mod *P*. As a consequence, in the following we will refer the positions *x^P ^*to as the *positions modulo P*. We shall use the terminology *site modulo P *as well.

#### Scoring function

**Figure 2:**
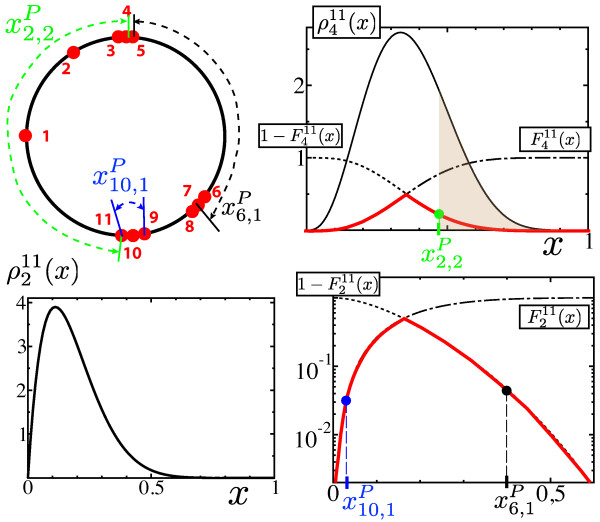
**Building elements for the scoring function (Eq. 3)**. *Left upper panel*: The sites modulo the period are numerated clockwise. Their relative positions depend on the value *P *of the period. The normalized (with respect to the period *P*) distance between the *j^th ^*nearest neighbors on each side of *i *is called $x_{i,j}^{P}$. *Right upper panel*: The *j^th ^*nearest neighbors on each side of any site *i *are separated by 2*j *- 1 sites. Hence, in order to compute the *p*-value $p_{j}^{N}\left(x_{i,j}^{P}\right)$ associated to any distance $x_{i,j}^{P}$, one needs the density distribution, respectively the repartition function, for the distances between sites that are separated by 2*j *- 1 sites, that is $\rho_{2j-1}^{N}$ and $F_{2j-1}^{N}$ respectively. For any *x *∈ 0[1], $p_{j}^{N}\left(x\right)$ corresponds to the area of the tails of $\rho_{2j-1}^{N}$ (indicated in brown for *N *= 11, *j *= 2 and $x=x_{2,2}^{P}$). For questions of computational readiness, $p_{j}^{N}\left(x\right)$ is approximated by $\min\left(F_{2j-1}^{N}(x),1-F_{2j-1}^{N}(x)\right)$ - see Eq. (4) - which is represented by the cuspate red curve. *Left lower panel*: The contribution of both $x_{10,1}^{P}$ and $x_{6,1}^{P}$ is evaluated from the same density function $\rho_{1}^{11}(x)$ since both distances correspond to sites that are separated by one single site. *Right lower panel*: $x_{10,1}^{P}$ corresponds to a dense region whereas $x_{6,1}^{P}$ reflects a poor region (see left upper panel).

The scoring function used here takes into account the self-information [17], or equivalently the information content, that is related to the distances separating the sites modulo *P*. More precisely, let us call *p*(*x^P^*) the *p*-value for any two such sites to be separated by a distance *x^P^*, supposing a random uniform distribution of the sites in {1, ..., *P*}. The scoring function then adds up the information contents [- log(*p*(*x^P^*))] of the nearest sites. Due to the presence of low *p*(*x^P^*)'s coming from both small and large distances *x^P^*, the presence of a *P*-periodic pattern results in a high scoring function. Geometrically speaking, small distances correspond to dense regions of the solenoid face view and large distances to poor regions (Fig. 2). To summarize, the scoring function at the core of the *SCM *consists of i) a modulo operation and ii) a cluster analysis of the resulting sites, which rewards both dense and poor regions. In geometrical terms, this can be viewed as i) a solenoidal projection and ii) a cluster analysis of the projected sites (Fig. 1).

#### Solenoidal spectra

The *SCM *consists, *in fine*, in computing the value of the scoring function (Eq. 3) at different periods *P*. It therefore consists in computing a spectrum, which is called hereafter the solenoidal spectrum (*SoS*). The presence of periodic patterns is then revealed by peaks in the spectrum that are exceptionally high. To quantitatively evaluate the likelihood of the peaks, the scores are interpreted in terms of a *p*-value. At a given period, this *p*-value corresponds to the probability of having a higher score by randomly drawing the sites according to a uniform law. The computation of the *p*-values generates a *p*-valued solenoidal spectrum (*pSoS*). In this regard, supposing that the spectrum is composed of *N_p _*independent peaks, the probability *p *to have more than one spectrum having at least one peak with a *p*-value lower than p* reads

(1) $$p=1-{\left(1-p^{*}\right)}^{{}^{N_{p}}}.$$

This allows to quantitatively evaluate the statistical significance of a *pSoS*, though the independence of the peaks, if any, may be a delicate point to prove. In any case, the probability for such a spectrum to occur by chance is lower than $1-{\left(1-p^{*}\right)}^{N_{p}}$.

### Identifying the periodic points

In a periodical dataset, all the sites are not expected to be positioned accordingly to the apparent periodicity. In particular, in addition to some wrongly predicted positions, datasets may contain sites that are generated from different sources or that belong to different families (*e.g*., different families of co-regulated genes). The positions of these sites are therefore not expected to be correlated. Interestingly, the *SCM *allows to determine which sites are concerned by a given periodicity. More precisely, a positional score can be defined for each site, which is related to the likelihood for the site to be periodically positioned with respect to the other sites of the dataset.

Given a period *P*, the positional score of any site *i *is calculated by analyzing the position of the nearest neighbors modulo *P*, *i.e*. the nearest neighbors on the *P*-solenoid face view. The principle consists in rewarding the sites that are located in the dense regions of the solenoid face view. To this end, we use, again, a quantity akin to the information content related to the distances of the nearest neighbors. Each pair of sites on each side of *i *is allocated with a probability *p_<_*for the sites to be separated by a distance that is inferior to their current distance, supposing the sites to be randomly drawn according to a uniform distribution. This leads to the information content [- log(*p_ <_*)]. Next, a scoring function $S^{\prime }(i,P)$ associated to *i *at the period *P *is defined. It is equal to the maximum information content obtained from the nearest neighbors, *i.e*. from the pair of nearest neighbors that has the lowest *p_ <_*.

The higher $S^{\prime }(i,P)$, the better the site is positioned according to the periodicity, or equivalently the denser the cluster to which it belongs on the solenoid face view (Fig. 1). The results are quantified by computing the *p*-value of $S^{\prime }$, hereafter referred to as *p_v_*($S^{\prime }$). In particular, the positional score of *i *at the period *P *is defined by:

(2) $$S_{pos}=-\log_{10}(p_{v}(S^{\prime }(i,P))).$$

## Implementation

### Periodic pattern detection: generating the solenoidal spectra

The next paragraph provides some details about the scoring function that is at the core of the *SCM*. The second paragraph provides further details about the computation of the *p*-values that are involved in the scoring function.

#### Scoring function

Let us consider the positions modulo *P *of a given set of *N *sites. Let us numerate the sites modulo *P *by sorting them clockwise (Fig. 2). For such a given site *i*, the normalized (with respect to the period *P*) distance between the two *j-*th nearest neighbors on each side of *i*, and hence separated by 2*j *- 1 sites, is noted $x_{i,j}^{P}$. We also call $p_{j}^{N}\left(x_{i,j}^{P}\right)$ the corresponding *p*-value for these sites to be separated by a distance $x_{i,j}^{P}$ - see next paragraph for the computation of the *p*-value, the information content associated to the measurement $x_{i,j}^{P}$ therefore reads $\left[-\log\left(p_{j}^{N}\left(x_{i,j}^{P}\right)\right)\right]$. The scoring function $S_{scs}(P)$ at the core of the *SoS *consists in summing up the information contents over the *J *first nearest neighbors around each of the *N *sites:

(3) $$S_{scs}(P)=-\frac{1}{2JN}\sum_{i=0}^{N-1}\sum_{j=1}^{J}\log\left(p_{i}^{N}\left(x_{i,j}^{P}\right)\right).$$

*J *represents the maximum number of nearest neighbors to be considered. Hence, the computation is all the faster that *J *is small. However, *J *must increase with *N *in order to efficiently detect dense regions. In this regard, all the reported results in this article have been obtained by choosing *J *= max(*E*[*N*/16], 1) where the function *E*[*x*] gives the integer part of *x*. We have observed that the precise dependence of *J *on *N *does actually not affect the detection.

#### p-values $p_{j}^{N}\left(x_{i,j}^{P}\right)$

For all *i *and *j*, $p_{j}^{N}\left(x_{i,j}^{P}\right)$ is the probability for generating a distance as extreme as $x_{i,j}^{P}$ when the sites are independently drawn according to a uniform law. In the case of dense regions, respectively poor regions, this corresponds to generate distances that are smaller, larger respectively, than $x_{i,j}^{P}$. This can be explicitly written in terms of the probability density $\rho_{2j-1}^{N}(x)$ of the random variable associated to the distance between any pair of sites that are separated by 2*j *-1 sites, which can be readily computed ∀*j *∈ {1,...,*N*/2} as explained now.

First, the probability $\rho_{i}^{N}(x)dx$ corresponds to finding one site at a distance *x *of a given site, and *i *sites at a distance lower than *x*. Next, there are *N *- 1 possibilities for placing one site at a distance *x *and $C_{N-2}^{i}$ for placing *i *of the remaining *N *- 2 sites, $C_{k}^{l}=\frac{l!}{k!(l-k)!}$ standing for the binomial factor. As a result, $\rho_{i}^{N}(x)$ reads

$$\rho_{i}^{N}(x)=(N-1)C_{N-2}^{i}x^{i}{\left(1-x\right)}^{N-2-i}.$$

For computational readiness, we use an approximation of the *p*-value that is valid for both short and large distances, which does not affect the issue of the *SCM *- see Fig. 2 for an illustrative explanation:

(4) $$p_{j}^{N}\left(x_{i,j}^{P}\right)≃\min\left(F_{2j-1}^{N}\left(x_{i,j}^{P}\right),\;\;1-F_{2j-1}^{N}\left(x_{i,j}^{P}\right)\right),$$

where $F_{2j-1}^{N}$ stands for the repartition function associated to $\rho_{2j-1}^{N}$, that is $F_{2j-1}^{N}(x)=\int_{0}^{x}dy\rho_{2j-1}^{N}(y)$. Dense regions correspond to small values of $F_{2j-1}^{N}\left(x_{i,j}^{P}\right)$ so that Eq. 4 leads to the right value of $p_{j}^{N}\left(x_{i,j}^{P}\right)$ in the limit of small distances, that is $p_{j}^{N}\left(x_{i,j}^{P}\right)=F_{2j-1}^{N}\left(x_{i,j}^{P}\right)$. On the other hand, poor regions correspond to values of $F_{2j-1}^{N}\left(x_{i,j}^{P}\right)$ close to 1 so that we also recover $p_{j}^{N}\left(x_{i,j}^{P}\right)=\left(1-F_{2j-1}^{N}\left(x_{i,j}^{P}\right)\right)$ in the limit of large distances. Intermediate values of $x_{i,j}^{P}$, *i.e*. close to the maximum of $\rho_{2j-1}^{N}$, do not play any crucial role for highlighting clusters, and hence, they are not crucial for the periodic detection method.

### Positional score

The positional score is calculated by analyzing the position of the nearest neighbors modulo *P*, *i.e*. the nearest neighbors on the *P*-solenoid face view. This means to compute the *p*-value *p*_<_for two sites to be separated by a distance that is inferior to their current distance supposing that the sites have been randomly drawn according to a uniform distribution.

Let us call $y_{i,j}^{P}$ the distance between any two sites modulo *P i *and *j*. The *p*_ <_'s are then given by:

$$p<(y_{i,j}^{P})=F_{(j-i+N)\%N}^{N}(y_{i,j}^{P})\;\forall i,j\in \{1,\ldots ,N\}$$

where % stands for the modulo operator. This leads to

$$S^{\prime }(i,P)=-\underset{{\langle j,k\rangle }_{J}}{\min}\left\{\log(p<(y_{j,k}^{P}))\right\}$$

where ⟨*j*, *k*⟩*_J _*stands for the set of pairs composed of two sites that lie on the *J *first nearest neighbors on each side of *i*.

## Results and discussion

### Periodic pattern detection

Two methods are often used to highlight the presence of periodic patterns. The first one is mostly used in the case of sparse boolean sequences, which is the case treated here. It consists in computing the histogram of the distances that separate each pair of points. The histogram is then analyzed thanks to a (discrete) Fourier transform. The second one is a standard procedure for analyzing continuous signals. It consists in computing an autocorrelation function, which is then analyzed thanks to a Fourier transform, too.

**Figure 3:**
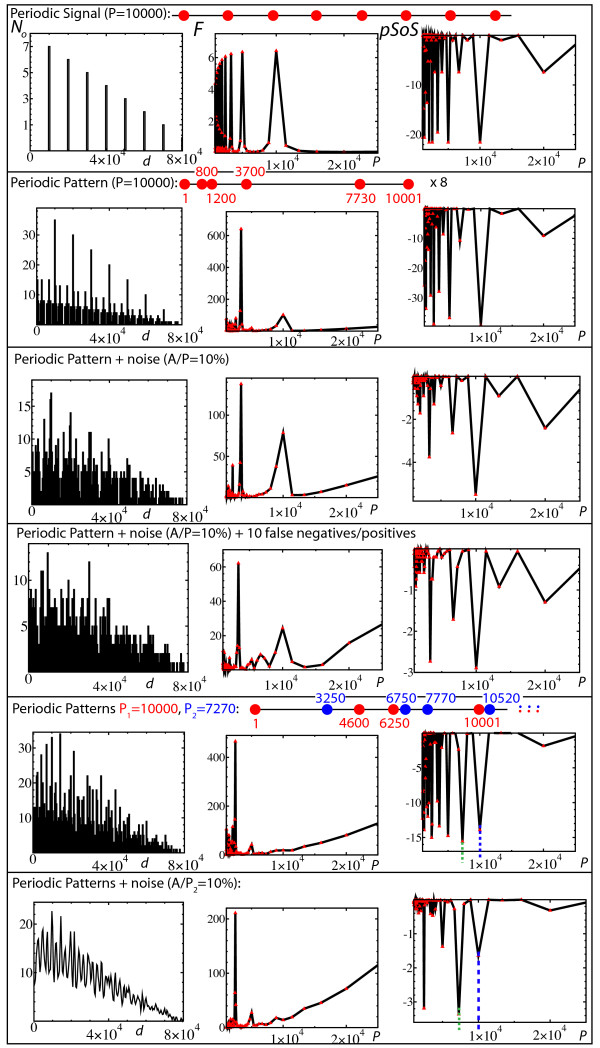
**Periodicity detection: Pair-distance histogram *vs*. Solenoidal Coordinate Method**. Each row corresponds to the analysis of a specific set of positions, which is indicated at the top of the row. The first column gives the pair-distance histogram, *i.e. *the number of occurrence *N*_o _of the distances (bin = 50). The second column gives the discrete Fourier transform *F *of the pair-distance histogram. Finally, the third column gives the *p*-value Solenoidal Spectrum (*pSoS*). See text for a detailed explanation of the results.

To illustrate the efficiency of the *SCM*, the *pSoS *is first compared to the pair-distance histogram technique for different kinds of small sets of positions (Fig. 3). Next, it is compared to the autocorrelation technique for site positions coming from both artificial and real datasets.

#### SCM versus pair-distance histograms - Fig. 3

Each row of Fig. 3 corresponds to the analysis of a specific set of positions, which is indicated at the top of the row. The first column gives the pair-distance histograms by reporting the number of occurrence *N*_*o *_of the distances (bin = 50). The second column gives the discrete Fourier transform *F *of the pair-distance histogram. Finally, the third column gives the *p*-value Solenoidal Spectrum (*pSoS*) using a semi-log scale. The first row shows the equivalence between *F *and *pSoS *for a Dirac comb with period *P *= 10000, *i.e*. a set of sites that are regularly spaced by a distance *P *= 10000. In both spectra, the peaks are harmonics of a main peak (period *P *= 10000). The second row shows the results for a set of positions that consists of a periodic succession (8 times here) of a complex pattern (red points). The period is still 10000. In *F*, the main peak is obtained at *P *~ 10000/6 whereas the *pSoS *still provides the main peak at *P *= 10000 (the other main peaks are harmonics of this period). In the third row, noise is added by drawing the positions according to a uniform distribution of amplitude *A*, which is centered around the sites of the second row (*i.e*., the second row corresponds to *A *= 0). For *A/P *= 10%, unlike the Fourier transform, most of the *pSoS*'s still provide a main peak at *P *= 10000. The fourth row shows the results for the same set of positions as in the third row, except that 10 points (of the 40 initial ones) have been deleted (false negatives) and replaced by 10 points at random locations (false positives). One can see that the *SCM *is still able to detect the presence of the periodic pattern, which demonstrates the robustness of the method with respect to data contamination.

The last two rows show the results for positions resulting from the combination of two periodic patterns having different periods (blue and red points). In the fifth row, positions correspond to a succession of the periodic motifs up to the position 80000, resulting in 56 points. The Fourier spectrum of the pair-distance histogram is flat around one of the main period (*P *= 10000) whereas all the peaks in the *pSoS *are harmonics of the two main periods *P *= 7270 and *P *= 10000, which are respectively indicated by the green and blue dashed vertical lines. The last row gives the curves that result from an average over 100 sets of positions drawn by adding noise to the previous case. In contrast to the Fourier spectrum of the pair-distance histogram, the two main periods are revealed by two sharp peaks in the *pSoS*, plus one main harmonic peak for each of them.

To summarize, the pair-distance histogram method is poorly efficient to highlight the periodic presence of a complex motif. More strikingly, the mixing of two motifs having two different periods lead to flat Fourier spectra of the pair-distance histograms around the expected periods. On the contrary, even in the presence of noise, the *pSoS *leads to well-defined peaks that clearly reveal the two different periods.

#### SCM versus autocorrelation function - Fig. 4

**Figure 4:**
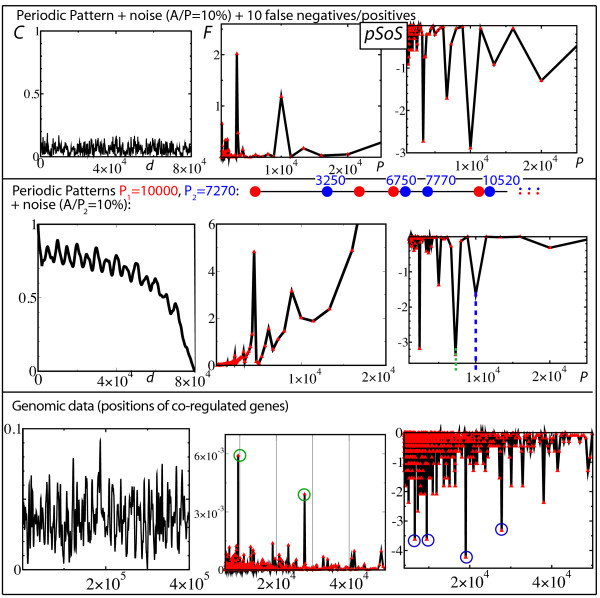
**Periodicity detection: Autocorrelation function *vs***. **Solenoidal Coordinate Method**. Each row corresponds to the analysis of a specific set of positions. The first (second) row corresponds to the case treated in the fourth (sixth) row of Fig. 3. The last row corresponds to the analysis of genomic data (see text). The first column gives the autocorrelation function *C*, the second column gives the discrete Fourier transform *F *of *C *and the third column gives the *p*-value Solenoidal Spectrum (*pSoS*) using a semi-log scale. See text for a detailed explanation of the results.

The autocorrelation function *C*(*x*) of an infinitely long sequence *X *is defined by $C(x)=\underset{L\to \infty }{\lim}\frac{N}{L}\sum_{i=0}^{L}X(i)\cdot X(i+x)$ where the normalization factor N is such that *C*(0) = 1. For small and sparse sequences, one needs first to smooth the sequence to avoid the product *X*(*i*) ·*X*(*i *+ *x*) to be mostly equal to 0. We call $\tilde{X}$ this smoothed sequence and further suppose $\tilde{X}$ to represent the best smoothing procedure for highlighting the periodic trend. In this case, the autocorrelation function is defined by $C(x)=\frac{N}{L-x}\sum_{i=0}^{L-x}\tilde{X}(i)\cdot \tilde{X}(i+x)$. The *SCM *is compared to the autocorrelation method in Fig. 4. The first (second) row corresponds to the case treated in the fourth (sixth) row of Fig. 3. The last row reports the analysis of positions coming from genomic studies.

Just as in the case of the pair-distance histogram technique, the two first rows demonstrate that autocorrelation functions are poorly efficient to highlight the periodic presence of complex motifs or the periodic presence of motifs having different periods. The results reported here have been obtained by smoothing the sequence using a 1000 long square window. Different smoothing procedures (Gaussian, different window sizes,...) can be checked to have little, if any, positive impact on the results.

In the last row, 90 genes of the 4639675 base-pair long *Escherichia coli *genome were analyzed. The positions were taken from the Regulon DataBase [18]. They correspond to the genes that present experimental evidence for being transcriptionally regulated by the transcription factor CRP, which is the transcription factor that regulates the most genes in *E. coli*. The Fourier transform of the autocorrelation function leads to two significant high peaks at periods 9508 and 27782 (green circles) whereas the *pSoS *leads to four significant high peaks at periods 6581, 9507, 19015 and 27782 (blue circles). In particular, the highest peak in the *pSoS*, i.e. the peak at the period 19015, has no counterpart in the autocorrelation function. These different results would lead to different interpretations of the genomic organization, and hence, to different predictions of the spatial organization of DNA [11].

### Positional scores

**Figure 5:**
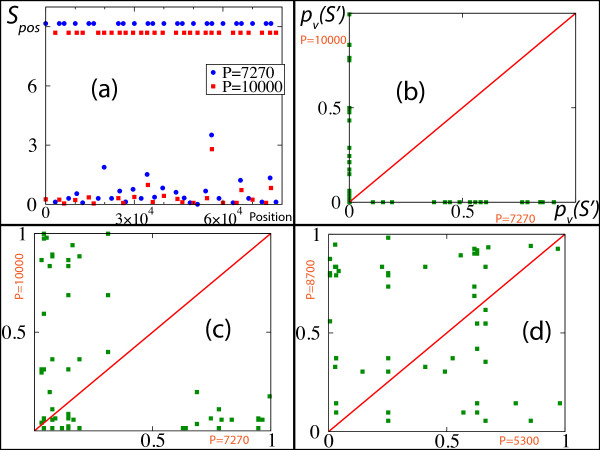
**Positional scores: detecting periodic sites**. (a): Positional score as a function of the position of the sites. The analyzed sequence is that of the fifth row of Fig. 3. (b): The x-axis, respectively the y-axis, is given by $10^{-S_{po\text{s}}(P)}=p_{v}(S^{\prime }(P))$ (see Eq. 2) computed at *P *= 7270, *P *= 10000 respectively. The analyzed sequence is that of (a). (c): Same plot as in (b) with some additional noise in the position of the sites (the parameters correspond to the last row of Fig. 3). (d): Same plot as in (c) but the x-axis is computed at *P *= 5300 and the y-axis at *P *= 8700. (b), (c), (d): the red lines give the bisector *f*(*x*) = *x*. See text for a detailed explanation of the results.

To illustrate the possibility to identify the periodic sites, we present in Fig. 5 two case studies in the situation of two periodic patterns having two different periods. These correspond to the case studies of the last rows of Fig. 3. Fig. 5a reports the positional score given by Eq. 2 as a function of the site position for noise-free periodic patterns (fifth row of Fig. 3). The blue (red) points give the positional scores of the points at the period 7270 (10000). High scores are obtained at period 7270, respectively 10000, for the points that form the 7270-periodic, 10000-periodic respectively, pattern.

A useful way for distinguishing the points that belong to different periodic trends then consists in plotting the quantity $10^{{}^{-S_{pos}(P)}}$, *i.e*. $p_{v}(S^{\prime }(P))$ in Eq. 2, computed at the period *P *= 10000 versus the same quantity computed at the period *P *= 7270, which is done in Fig. 5b. In this plot, one can clearly distinguish the points that belong to the 10000-periodicity (points along the *x*-axis) from those that belong to the 7270-periodicity (points along the *y*-axis). Interestingly, this representation also allows to distinguish the different points for a sequence that is distorted. In Fig. 5c, the distortion consisted in adding noise to the sequence used in Fig. 5a and 5b. This was done by drawing the positions according to a uniform distribution of amplitude 727 (*i.e*. 10% of 7270), which is centered around the original sites. This hence corresponds to the situation of the last row of Fig. 3. In contrast, in Fig. 5d, we report the quantity 10^-*Spos*(*P*) ^computed at *P *= 8700 versus the same quantity computed at *P *= 5300, *i.e*. at periods where no regularities are expected. In this situation, the points are no more separated.

## Conclusion

Pair-distance histograms and auto-correlation functions, either analyzed by discrete or continuous Fourier transforms, may be poorly appropriate for highlighting the presence of periodic patterns in sparse and noisy sequences. More importantly, both methods do not succeed in disentangling multiple patterns having different periods so that the corresponding Fourier spectra are flat at the periods supposedly characterizing the sequence (Fig. 3 and 4). In contrast, the solenoid coordinate method (*SCM*) has been built in order to be particularly sensitive to any periodic patterns, even in the case of overlapping patterns with different periods. Its robustness to signal distortion, which can be due to the presence of noise, false positives or/and false negatives, stems from the remarkable alignment properties of periodic sites when they are represented in a solenoidal coordinate system with the right period (Fig. 1). It must also be noted that the *SCM *does not need any smoothing of the original sequence as in the case of the autocorrelation function. Finally, thanks to the definition of a positional score, we have shown that the *SCM *framework further allows to identify the sites that participate most in a periodic tendency. This should be particularly useful for identifying periodic genes, and hence, for investigating their functional properties.

The present method is suited to sparse (boolean) sequences that contain a rather small number of sites (1's). More precisely, the computational time for running a spectrum of a sequence containing *N *sites scales as *JN *~ *N*^2 ^(see Eq. 3). The method is therefore poorly scalable in its present form. Different improvements along this direction can be contemplated. A possible one would consist in computing the Kullback-Leibler divergence (with respect to a uniform distribution) of the density distribution of the sites modulo the periods, *i.e*. the Kullback-Leibler divergence of the density distribution along the solenoid face views. This cannot be done when the number of sites is too small, which was the case treated here.

## Availability and requirements

The software is available for public use at http://www.issb.genopole.fr/MEGA/Softwares/iSSB_SolenoidalApplication.zip.

## List of abbreviations used

*SCM*: solenoidal coordinate method; *SoS*: solenoidal spectrum; *pSoS*: *p*-valued Solenoidal Spectrum

## Competing interests

The authors declare that they have no competing interests.

## Authors' contributions

IJ, JH and FK participated in the design of the study. IJ and JH performed the statistical analysis. IJ, JH and FK wrote the paper. All authors have read and approved the final manuscript.
